# ‘Hot cross bun’ sign with leptomeningeal metastases of breast cancer: a case report and review of the literature

**DOI:** 10.1186/s12957-015-0483-z

**Published:** 2015-02-12

**Authors:** Zhenyu Pan, Guozi Yang, Tingting Yuan, Yongxiang Wang, Xiaochuan Pang, Yan Gao, Lihua Dong

**Affiliations:** Department of Radiotherapy, the First Hospital of Jilin University, 71 Xinmin Street, Changchun, 130021 China; Department of Radiology, Norman Bethune First Hospital, Jilin University, 71 Xinmin Street, Changchun, 130021 China; Department of Clinical Laboratory, Norman Bethune First Hospital, Jilin University, 71 Xinmin Street, Changchun, 130021 China

**Keywords:** Breast cancer, Hot cross bun, Leptomeningeal metastases

## Abstract

**Background:**

The ‘hot cross bun’ (HCB) sign refers to a cruciform-shaped hyperintensity within the pons found on T2-weighted magnetic resonance imaging (MRI). It is commonly associated with atrophy of the pons, cerebellum, and putamen in multiple system atrophy (MSA). In this report, we describe a rare case of the HCB sign in an adult female patient with leptomeningeal metastases of breast cancer without any signs of brain atrophy.

**Case presentation:**

The patient was a 58-year-old woman diagnosed with grade 2 ductal breast carcinoma, who had undergone a right mastectomy, followed by chemotherapy treatments and chest wall radiotherapy. The tumor had metastasized to the skin, and the patient presented with vomiting, drowsiness, and intermittent episodes of confusion, slurred speech, and involuntary movements. Immunohistochemical staining demonstrated a triple-negative status of the tumor. Axial T1-weighted MRI showed a linear enhancement in the cerebellar sulcus. A diagnosis of leptomeningeal metastases of breast cancer was confirmed by detection of tumor cells in the cerebrospinal fluid. Axial T2-weighted MRI indicated a cruciform hyperintensity in the pons without any atrophy of the pons, cerebellum, or putamen.

**Conclusion:**

The HCB sign can occur with leptomeningeal metastases of solid tumors, though the underlying mechanisms remain unknown.

## Background

The ‘hot cross bun’ (HCB) sign is typically seen as a cruciform hyperintensity in the pons on T2-weighted magnetic resonance imaging (MRI) of the brain. It is commonly associated with atrophy of the pons, cerebellum, and putamen [1], due to a selective loss of transverse pontocerebellar fibers and neurons of the pontine raphe, with preservation of corticospinal tracts and tegmental neurons [2]. Although the HCB sign is seen more often in multiple system atrophy (MSA), its appearance has also been associated with brain atrophy in a variety of neurodegenerative diseases, such as cerebrotendinous xanthomatosis [3], human immunodeficiency virus-related progressive multifocal leukoencephalopathy [4], variant Creutzfeldt-Jakob disease [5], spinocerebellar ataxia [6], and with Parkinsonism secondary to presumed vasculitis, where it is thought to reflect Wallerian degeneration of transverse pontocerebellar fibers [7]. The mechanisms underlying the HCB sign remain unclear and likely differ among the various diseases.

Here, we report a rare case of the HCB sign in a patient with breast cancer leptomeningeal metastases who showed no detectable signs of brain atrophy. We also explore the pathogenesis of the HCB sign.

## Case presentation

A 58-year-old woman previously diagnosed with breast cancer presented to our hospital with a 7- to 10-day history of progressively worsening intermittent headaches, vomiting, drowsiness, episodes of confusion, slurred speech, and involuntary movements. The patient had undergone a right mastectomy for a breast tumor 18 months earlier. The tumor size (3.7 × 2.5 × 2.0 cm) and type (grade 2 invasive ductal breast carcinoma) were determined by postoperative pathologic analysis. In addition, the tumor was surrounded by a large area of necrotic tissue, exhibited infiltrative growth, and was infiltrated with lymph cells (in a scattered pattern). Vascular thrombosis and right axillary lymph node metastasis (5/15) were observed. Immunohistologic staining demonstrated a triple-negative status (for estrogen and progesterone receptors, and human epidermal growth factor receptor 2) and a high (60%) Ki-67 percentage. The patient underwent six 21-day cycles of adjuvant chemotherapy with docetaxel (140 mg q.d.) and tetrahydropyranyl adriamycin (70 mg b.i.d.) followed by chest wall radiotherapy. The patient exhibited skin redness, ulceration, and a painful purulent discharge on the right chest wall 4 months after the radiation therapy. Subsequently, the size of the lesion gradually increased. Skin biopsy and pathologic analysis indicated metastatic carcinoma of breast cancer. The patient received five 21-day cycles of chemotherapy with paclitaxel (150 mg q.d.) and carboplatin (300 mg q.d.). After three of these cycles, the size of the lesion had reduced slightly. Upon review, the clinical response to chemotherapy was evaluated as a stable disease.

**Figure 1:**
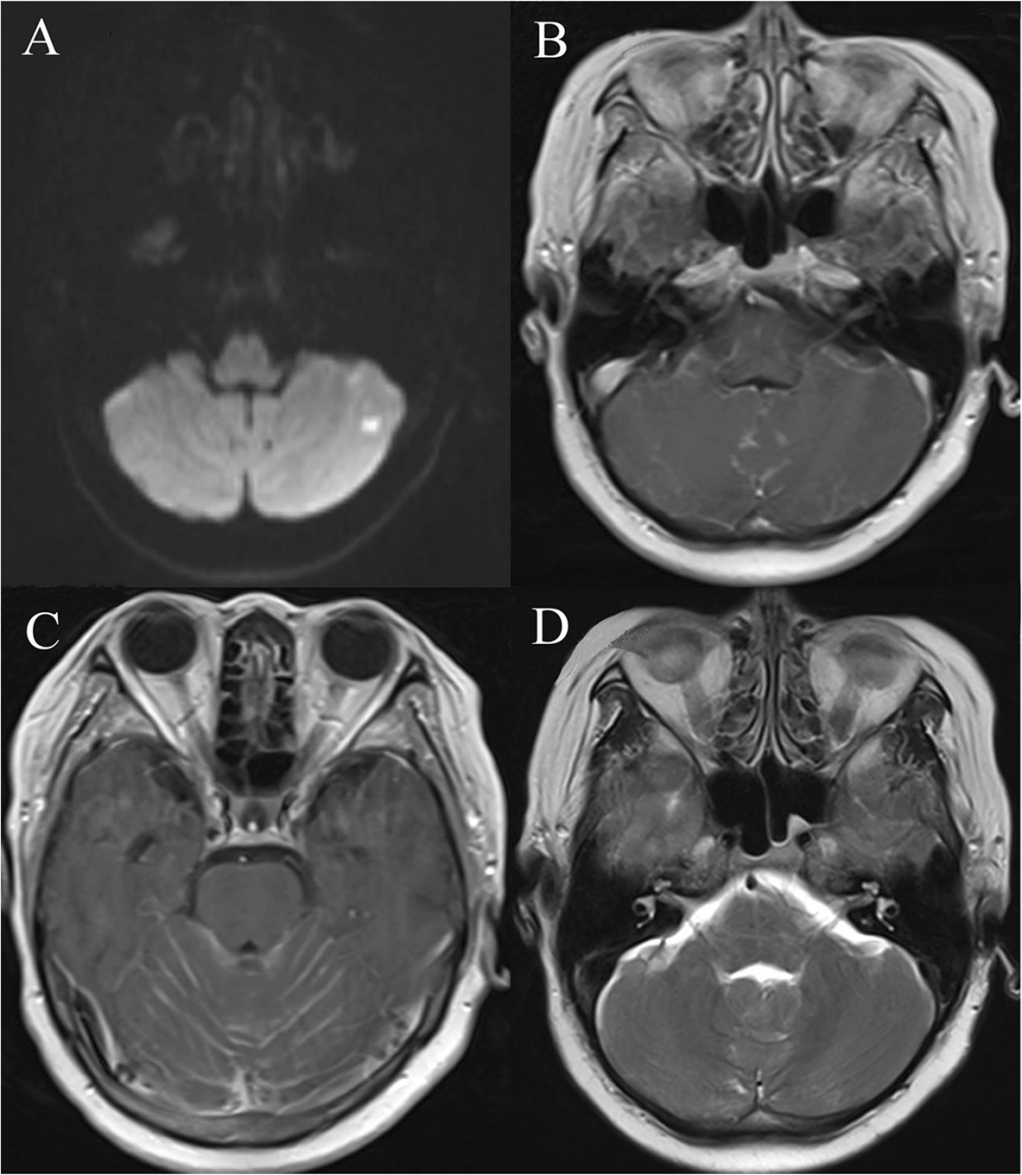
**Magnetic resonance imaging (MRI) revealed the hot cross bun sign. (A)** Diffusion-weighted MRI showing a patchy hyperintensity in the left cerebellar hemisphere; no enhancement was observed on contrast-enhanced MRI **(B)**, suggesting cerebral infarction. **(C)** Axial T1-weighted MRI showing diffuse linear enhancement in the cerebellar sulcus, suggesting leptomeningeal metastases of breast cancer. **(D)** Axial T2-weighted MRI showing a cruciform hyperintensity in the pons; no atrophy was observed in the pons or cerebellum. All contrast-enhanced imaging was carried out with gadolinium-based contrast agents.

The patient was presented at our clinic 2 weeks after the last chemotherapy dose and was admitted to our hospital. History taking at intake did not reveal any chronic diseases, and she was confused and uncooperative during the initial physical examination. A computed tomography scan of the head demonstrated no abnormalities. Diffusion-weighted MRI exhibited a patchy hyperintensity in the bilateral cerebellar hemispheres (Figure 1A), but no contrast enhancement was observed on contrast-enhanced MRI (Figure 1B). Axial T1-weighted MRI indicated a diffuse linear enhancement in the cerebellar sulcus, suggesting leptomeningeal metastases of breast cancer (Figure 1C). A cruciform-shaped hyperintensity appeared in the pons on axial T2-weighted MRI (Figure 1D). There were no abnormalities of the ventricular system or shift of the midline structures observed.

**Figure 2:**
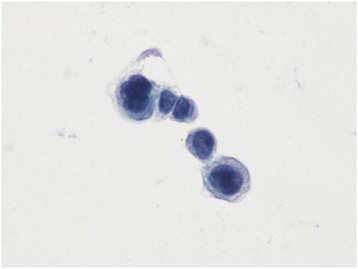
**Presence of tumor cells present in the cerebrospinal fluid.** A papanicolaou-stained, liquid-based, thin-layer preparation of cerebrospinal fluid is shown (400×). Tumor cells were of various large sizes, appeared clustered together, and had abnormal ratios of nucleus (dark blue) to cytoplasm (light blue). The features suggested adenocarcinoma cells.

The patient was diagnosed with lacunar infarction in bilateral cerebellar hemispheres and leptomeningeal metastases from breast cancer. A lumbar puncture was performed, followed by cerebrospinal fluid (CSF) analysis, which revealed a CSF pressure of 380 mm H_2_O. Total protein, glucose, and chloride concentrations were 0.69 g/L (normal range: 0.15 to 0.45 g/L), 2.11 mmol/L (normal range: 2.3 to 4.3 mmol/L), and 109.9 mmol/L (normal range: 119 to 129 mmol/L), respectively. Moreover, breast cancer cells were detected in the CSF following cytologic evaluation of the fluid (Figure 2). The disease rapidly progressed, and the patient went into a shallow coma with unequal pupil size, left lower extremity muscle strength of 3/5, and a positive pathologic reflex. The patient remained in a coma state (Glasgow Coma Scale score: 9) the day after her lumbar puncture and was discharged without any treatment.

### Discussion

Mechanisms have been proposed to explain the appearance of the HCB sign, including selective degeneration of pontine fibers in MSA [2], Parkinsonism secondary to presumed vasculitis [7], and cerebrotendinous xanthomatosis [3]. In addition, gliosis in pontocerebellar fibers in the middle part of the reticular formation combined with neuronal loss can produce the HCB sign on T2-weighted MRI [8]. However, the HCB sign was observed in the present case in the absence of any apparent cerebellar, pontine, or putaminal atrophy, indicating it can also appear in other diseases, such as leptomeningeal metastases from breast cancer.

Zhang et al. [9] reported the HCB sign in a case of malignant melanoma, where the patient had a history of epilepsy since childhood and exhibited signs of atrophy in the pons and cerebellum. The appearance of the HCB sign in that case may have been a result of epilepsy-associated vasospasm and neuronal hypoxia. In the current case, however, the patient had no prior history of any chronic diseases. To date, there are no other reports in the publicly available literature on HCB signs in patients with primary tumors or secondary brain tumors. The patient in the current case was diagnosed with breast cancer leptomeningeal metastases based on her history of breast cancer, neuroradiologic findings, clinical manifestations, and results of CSF cytologic analysis, which represent the gold standard for diagnosis [10]. Leptomeningeal metastases are from infiltration of tumor cells into the pia or arachnoid mater and their subsequent diffusion into the subarachnoid space. They are considered rare, devastating complications of malignant solid tumors. The short median survival time of patients with leptomeningeal metastases of solid tumors may be insufficient for the development a chronic disease that leads to atrophy in the pons.

Based on the lack of signs of atrophy, we hypothesize that the mechanism resulting in the HCB sign in the present case differs from previous cases and may have been due to neuronal damage caused by cerebral ischemia, neuronal nutritional deficiencies, metabolic abnormalities, and/or direct tumor invasion. For example, leptomeningeal metastases from solid tumors can lead to many pathophysiologic changes, including: 1) hydrocephalus or increased intracranial pressure due to blockade of CSF flow; 2) neurologic dysfunction caused by tumor compression or invasion of the brain and spinal cord; 3) disrupted blood-CSF barrier; 4) tumor compression-induced brain ischemia from the invasion of blood vessels on the brain convexity and in the Virchow-Robin space, which can cause vascular spasms, encephalopathy, and reduced blood supply [11]; and 5) metabolic disorder due to competitive inhibition of cerebral glucose utilization by the neuron [12].

## Conclusions

In conclusion, this case report describes a rare appearance of the HCB sign in a patient with leptomeningeal metastases of breast cancer, thus demonstrating that its appearance is not limited to conditions characterized by regional atrophy. The mechanisms underlying the HCB signs are complex and remain to be explored in future studies.

## Consent

Written informed consent was obtained from the patient and her parents for publication of this case report and any accompanying images. A copy of the written consent is available for review by the series editor of this journal.

## Abbreviations

CSF: cerebrospinal fluid
HCB: hot cross bun
MRI: magnetic resonance imaging
MSA: multiple system atrophy
